# Exploring spatial variations and factors associated with skilled birth attendant delivery in Ethiopia: geographically weighted regression and multilevel analysis

**DOI:** 10.1186/s12889-020-09550-3

**Published:** 2020-09-23

**Authors:** Achamyeleh Birhanu Teshale, Adugnaw Zeleke Alem, Yigizie Yeshaw, Sewnet Adem Kebede, Alemneh Mekuriaw Liyew, Getayeneh Antehunegn Tesema, Chilot Desta Agegnehu

**Affiliations:** 1 Department of Epidemiology and Biostatistics, Institute of Public Health, College of Medicine and Health Sciences, University of Gondar, Gondar, Ethiopia; 2 Department of Physiology, School of Medicine, College of Medicine and Health Sciences, University of Gondar, Gondar, Ethiopia; 3 School of Nursing, College of Medicine and Health Sciences and Comprehensive Specialized Hospital, University of Gondar, Gondar, Ethiopia

**Keywords:** Skilled birth attendant delivery, Geographically weighted regression, Multilevel analysis, Ethiopia

## Abstract

**Background:**

Skilled birth attendant (SBA) delivery is vital for the health of mothers and newborns, as most maternal and newborn deaths occur at the time of childbirth or immediately after birth. This problem becomes worsen in Ethiopia in which only 28% of women give birth with the help of SBA. Therefore, this study aimed to explore the spatial variations of SBA delivery and its associated factors in Ethiopia.

**Methods:**

A secondary analysis was carried out using the 2016 Ethiopian Demographic and Health Survey. A total weighted sample of 11,023 women who had a live birth in the 5 years preceding the survey was included in the analysis. Arc-GIS software was used to explore the spatial distribution of SBA and a Bernoulli model was fitted using SaTScan software to identify significant clusters of non-SBA delivery. The Geographic Weighted Regression (GWR) was employed in modeling spatial relationships. Moreover, a multilevel binary logistic regression model was fitted to identify factors associated with SBA delivery.

**Results:**

In this study, SBA delivery had spatial variations across the country. The SaTScan spatial analysis identified the primary clusters’ spatial window in southeastern Oromia and almost the entire Somalia. The GWR analysis identified different predictors of non- SBA delivery across regions of Ethiopia. In the multilevel analysis, mothers having primary and above educational status, health insurance coverage, and mothers from households with higher wealth status had higher odds of SBA delivery. Being multi and grand multiparous, perception of distance from the health facility as big problem, rural residence, women residing in communities with medium and higher poverty level, and women residing in communities with higher childcare burden had lower odds of SBA delivery.

**Conclusion:**

Skilled birth attendant delivery had spatial variations across the country. Areas with non-skilled birth attendant delivery and mothers who had no formal education, not health insured, mothers from poor households and communities, Primiparous women, mothers from remote areas, and mothers from communities with higher childcare burden could get special attention in terms of allocation of resources including skilled human power, and improved access to health facilities.

## Background

Globally, maternal mortality ratio had decreased from 385 in 1990 to 216 deaths per 100,000 live births in 2015. Despite this global decrement, there is still high rates of maternal mortality in sub-Saharan Africa and South Asia; which accounts for 88% of worldwide maternal deaths [1]. In Ethiopia, maternal mortality ratio declines from 871 per 100,000 live births in 2000 to 412 per 100,000 live births in 2016. Despite this decrement, the plan of reducing the maternal mortality ratio to 199 maternal deaths per 100,000 live births by 2020 is still unachieved [2–5].

The Sustainable Development Goal planned to decrease maternal mortality ratio to 70 deaths per 100,000 live births by 2030 and one of the best ways to achieve this objective is through providing skilled and respectful care during delivery [6–8].

Skilled birth attendant (SBA) delivery is vital for the health of mothers and her newborn, as most maternal and newborn deaths occur around the time of childbirth or immediately after birth [9–13]. Even though the World Health Organization (WHO) recommends SBA to be present at every birth, whether the birth occurs in a facility or at home, millions of births occur annually without the assistance of SBA [14, 15].

Eighty percent of live births in the world and 59% of births in sub-Saharan Africa are attended by skilled health personnel [16]. This prevalence of SBA delivery in sub-Saharan Africa also varies across countries ranging from 13% in Nigeria to 77% in Kenya [17–20]. In Ethiopia, only 18.8 to 29.2% of births are attended by skilled health personnel [2, 21, 22].

Skilled birth attendant delivery is associated with the age of the mother [23, 24], education of the mother [25, 26], parity [27], birth order [28], religion [29, 30], health insurance coverage [30], distance to the health facility [23, 31, 32], residence, community level of women education, community poverty level, and community level of media exposure [27, 33–35].

Though SBA delivery depends on both individual and community level factors, to the best of our knowledge limited studies have been done beyond individual level factors to consider the community level factors affecting SBA delivery in Ethiopia. Besides, previous studies did not consider the spatial distribution of SBA delivery and spatial regression analysis, which are important for policymakers for the proper allocation of scarce resources in poor clinical setups like Ethiopia. Therefore, this study aimed to determine the spatial variations of SBA delivery and its associated factors. The findings of this study might give insight for policymakers, researchers, and health professionals about the status of SBA delivery in the country, to take targeted interventions for those regions with a low prevalence of SBA delivery.

## Methods

### Study area, data source, and population

We used the Ethiopian Demographic and Health Survey (EDHS) 2016 to conduct this study. The EDHS is a survey collected across the nine regional states and two city administrations of Ethiopia every 5 years. The latest EDHS (EDHS 2016), was conducted from January 18, 2016, to June 27, 2016. The sample was stratified and selected in two stages. In the first stage, a total of 645 EAs (Enumeration Areas) (443 in rural areas) were selected with probability proportional to EA size (using 84,915 EAs created for the 2007 Ethiopian population and housing census as a sampling frame). A fixed number of 28 households per cluster were selected in the second stage with an equal probability systematic selection after the household listing was done in all of the selected EAs (the lists of households used as a sampling frame for the selection of households in the second stage). Any additional information about data collection, sampling, and questionnaires used in the surveys are described in detail in the 2016 EDHS report [2].

For our study, women aged 15 to 49 years who gave birth within 5 years preceding the survey were included. For those women with two or more live births during the preceding 5 years, data from the most recent birth was used. Accordingly, a total weighted sample of 11,023 women was used in the final analysis.

### Variables of the study

The outcome variable for this study was delivery by SBA. Skilled attendant delivery in this study refers to births delivered with the assistance of doctors, nurses/midwives, health officers, and health extension workers [2].

After searching of literatures, both individual and community level factors were incorporated as independent variables (for the multilevel analysis). The individual level factors include maternal education, maternal age, religion, parity, birth order, household wealth status, access to mass media, and health insurance coverage. The community-level factors were community level of women education, community poverty level, community level media exposure, community childcare burden, perception of distance to the health facility, region, and place of residence (Table 1).

**Table 1:** Definition/description and measurement of independent [both individual and community level] variables

Variables	Definition/description
Individual level factors	
Maternal education	Re-coded as “0” for no formal education, “1” for primary education, and “2” for secondary and above (combining secondary and higher education categories).
Maternal age (Years)	Re-coded into three categories with values of “0” for 15–24, “1” for 25–34, and “2” for 35–49.
Religion	Re-coded in four categories with a value of “0” for Orthodox Christian, “1” for Muslim, “2” for protestant, and “3” for other religious groups (Catholic, traditional, and the other religious categories).
Parity	Re-coded as “0” for Primiparous (having parity of one), “1” for multiparous (having parity of 2–4), and “2” for grand multiparous (having parity of 5 and above).
Birth order	Re-coded into four categories [“0” for 1st order birth, “1” for 2nd order birth, “2” for 3rd order birth, and “3” for 4th order and above birth].
Household wealth status	It was coded as “0” for the poorest, “1” for poorer, “2” for middle, “3” for richer, and “4” for richest in the data set and we used as it is for our analysis.
Media exposure	Created by combining whether a respondent reads a newspaper, listen to the radio, and watch television and coded as “0” no (if women were not exposed to at least one of the media) and “1” for yes (if a woman had exposed to at least one of these media).
Health insurance coverage	Re-coded as “0” if a woman was not covered by health insurance and “1” if a woman was covered by health insurance.
Community level factors	
Perception of distance from the health facility	Re-coded, as “0” if a woman perceives distance from the health facility as a big problem and “1” if a woman perceives distance from the health facility as not a big problem.
Residence	Re-coded as “0” for urban and “1” for rural residence
Region	From the data set region was coded as “0–10” for Afar, Tigray, Amhara, Oromia, Somalia, Benishangul, SNNPR, Gambela, Harari, Addis Ababa, and Dire Dawa respectively and we used as it is for this study.
Community level of women education	Measured by the proportion of women with a minimum of primary level of education derived from data on mothers or respondents’ level of education and coded as: “0″ for low (communities in which < 50% women had at least primary education) and “1″ for high (communities in which ≥50% women had at least primary education) community level of women education.
Community level of child care burden	Measured by the proportion of women who had five or more children derived from data on the total number of children ever born. It was coded as “0” for low (communities in which < 25% women had five or more children), “1” for medium (communities in which 25–50% women had five or more children), and “2” high (communities in which ≥50% women had five or more children) community level childcare burden.
Community poverty level	Measured by the proportion of women in the poorest and poorer quintiles derived from data on wealth index. It was coded as “0” for low (communities in which < 25% women had poorest and poorer wealth quintiles), “1” for medium (communities in which 25–50% women had poorest and poorer quintiles), and “2” for higher (communities in which ≥50% women had poorest and poorer wealth quintiles) poverty communities.
Community level media exposure	Measured by the proportion of women who had at least exposed to one media, either television, radio, or newspaper. It was coded/categorized in a similar way to that of the above community level variables as “0” for low, “1” for medium, and “2” for high community level media exposure.

The above four community level factors [community level of women education, community poverty level, community level media exposure, and community level of child care burden] were not directly found in the EDHS data. As a result, they were created by aggregating their respective individual level factors (Table 1).

Moreover, different explanatory variables were considered in modeling spatial relationships. The candidate variables were proportions of women with no education, the proportion of Primiparous women, proportion of women from poor household wealth status, proportion of women with no health insurance, and proportion of women who perceives distance from the health facility as a big problem, and proportion of women with no media exposure.

### Data management and statistical analysis

#### Spatial analysis

Both Arc GIS version 10.3 and Kuldorff’s SaTScan version 9.6 software were used to explore the spatial distribution of SBA and to identify significant hotspot areas/clusters of non-SBA respectively. The global spatial autocorrelation was done using the Global Moran’s I statistic, which is used to ascertain whether the spatial distribution of SBA is clustered, dispersed, or random across the country [36, 37].

The spatial interpolation technique was employed to predict the prevalence of non-SBA delivery on the un-sampled/unmeasured areas based on the sampled measurements. The Kriging spatial interpolation method was used in this study for predicting non-SBA in unobserved areas since it had a small mean square error and residual. In addition, hot spot and cold spot analysis were done to identify specific significant hot spots areas (areas with higher rates of non-SBA delivery) and cold spot areas (areas with lower rates of non-SBA delivery) using Getis-Ord Gi* statistics, relative to the mean SBA rate across the country.

Moreover, we conducted a spatial scan statistical analysis to identify significant primary and secondary clusters. In the SaTScan analysis, the Bernoulli based spatial scan statistical analysis which requires information about the location of a set of cases (deliveries that were not attended by SBA) and controls (those who delivered by SBA), as well as the coordinate files (latitude and longitude) was used. The default maximum spatial cluster size of < 50% of the population was used as an upper limit for detecting both small and large clusters and ignored clusters that contained more than the maximum limit. The primary and secondary clusters were identified and *p* values were assigned and ranked using their LLR test based on the 999 Monte Carlo replications. The circle with the highest LLR test statistic was defined as the most likely (primary) cluster, the cluster that is least likely to have occurred by chance. For each identified cluster, the location, radius/size, log-likelihood ratio (LLR) test statistic with its *p*-value, and the relative risk (RR) were reported. The RR represents how much more common non-SBA delivery with a value of greater than one is used to indicate an increased risk of non-SBA delivery in a specified spatial window as compared to outside the window.

#### Spatial regression (modeling spatial relationships)

We also used Arc GIS version 10.3 for assessing spatial relationships/spatial regression. Spatial regression modeling was performed to identify predictors of the observed spatial patterns of non-SBA delivery. We conducted both ordinary least square (OLS) and geographically weighted regression (GWR) analysis. Findings from ordinary least squares (OLS) regression are only reliable if the regression model satisfies all of the assumptions that are required by this method. While conducting the OLS regression the assumptions to be fulfilled, the model performance, as well as the model significance were checked [38, 39]. In addition, a certain independent variable may be a strong predictor in one cluster and it may not be in another cluster. This is non-stationarity and can be identified using GWR [40–42]. These two (OLS and GWR) models were compared using different parameters. Finally, the coefficients which were created using GWR were mapped.

#### Multilevel analysis

Stata 14 software was used for analysis. To avoid geographical strata selection variability and non-responses, as well as to assure representativeness and have better estimations of parameters, sampling weight was done throughout our analysis. Both bivariable and multivariable multilevel logistic regression analyses were performed. Because of the hierarchical nature of EDHS, we used the multilevel logistic model for the appropriate estimation of parameters. To do so, four models have been fitted; the null model- a model without explanatory variables, the model I- a model with individual-level factors only, model II- a model with community-level factors, and model III- a model with both individual and community-level factors simultaneously. Among the four fitted models, the model with the lowest deviance was selected as the best-fitted model. The intraclass correlation coefficient (ICC), a proportional change in variance (PCV), and median odds ratio (MOR) were also used to examine the clustering effect and the extent to which community-level variability explains the unexplained variance of the null model.

## Results

### Background characteristics of study participants

A weighted sample of 11,023 women aged 15 to 49 years was included in this study (Table 2). The median age of the participants was 28 (IQR = 25–34) years. A large proportion of study participants, 7284 (66.08%) had no formal education., Majority, 10,633 (96.46%) of the study participants were not covered by Health Insurance. While measuring the characteristics of the community, 9807 (88.97%) of the women were rural dwellers. From the total study subjects, only 2526 (25.26%) were from the lower community poverty level and 6243 (56.64%) were from a higher proportion of community media exposure. Most, 4851(44.01%) and 2296 (20.83%) of study participants were from Oromia and Southern Nations, Nationalities and Peoples’ Region (SNNPR) respectively. About half, 5652 (51.28%) of the study participants were living from communities with a higher level of women education. Regarding community childcare burden, 6194 (56.19%) of the participants were living in a community of higher childcare burden. The majority, 6677 (60.57%) of study participants perceive distance from the health facility as a big problem (Table 2).

**Table 2:** Background characteristics of study participants, Ethiopia, EDHS 2016

Variables	Frequency [***N*** = 11,023]	Percentage
Maternal age (years)		
15–24	2446	22.19
25–34	5843	53.00
35–49	2734	24.84
Maternal education		
No formal education	7284	66.08
Primary	2951	26.77
Secondary and above	788	7.15
Religion		
Orthodox Christian	3772	34.22
Muslim	4561	41.38
Protestant	2329	21.13
Other^a^	361	3.27
Parity		
Primiparous	1434	13.01
Multiparous	4836	43.87
Grand multiparous	4753	43.12
Birth order		
1st order	2058	18.67
2nd order	1784	16.18
3rd order	1575	14.29
4th and above	5606	50.83
Covered by health insurance		
Yes	390	3,54
No	10,633	96.46
Wealth index		
Poorest	2636	23.92
Poorer	1520	22.86
Middle	2280	20.68
Richer	1999	18.13
Richest	1588	14.41
Media exposure		
No	7376	66.91
Yes	3647	33.09
Perception of distance from Health facility		
Big problem	6677	60.57
Not a big problem	4346	39.43
Region		
Tigray	716	6.49
Afar	114	1.04
Amhara	2072	18.80
Oromia	4851	44.01
Somali	508	4.61
Benishangul	122	1.10
SNNPR	2296	20.83
Gambela	27	0.24
Harari	26	0.23
Addis Ababa	244	2.21
Dire Dawa	47	0.43
Residence		
Urban	1216	11.03
Rural	9807	88.97
Community-level of women education		
Low	5371	48.72
High	5652	51.28
Community poverty level		
Low	2784	25.26
Medium	4266	38.70
High	3973	36.05
Community childcare burden		
Low	2214	20.09
Medium	2615	23.72
High	6194	56.19
Community-level media exposure		
Low	1731	15.70
Medium	3049	27.65
High	6243	56.64

^a^ = Catholic, Traditional, Other, N = Total sample size (weighted)

### Spatial analysis of non-SBA delivery

#### Spatial autocorrelation

The global spatial autocorrelation analysis revealed a clustering pattern of women’s non-SBA delivery across Ethiopia (Global Moran’s I = 0.439, *p* value< 0.001).

#### Spatial distribution and interpolation of non-SBA delivery

**Fig. 1:**
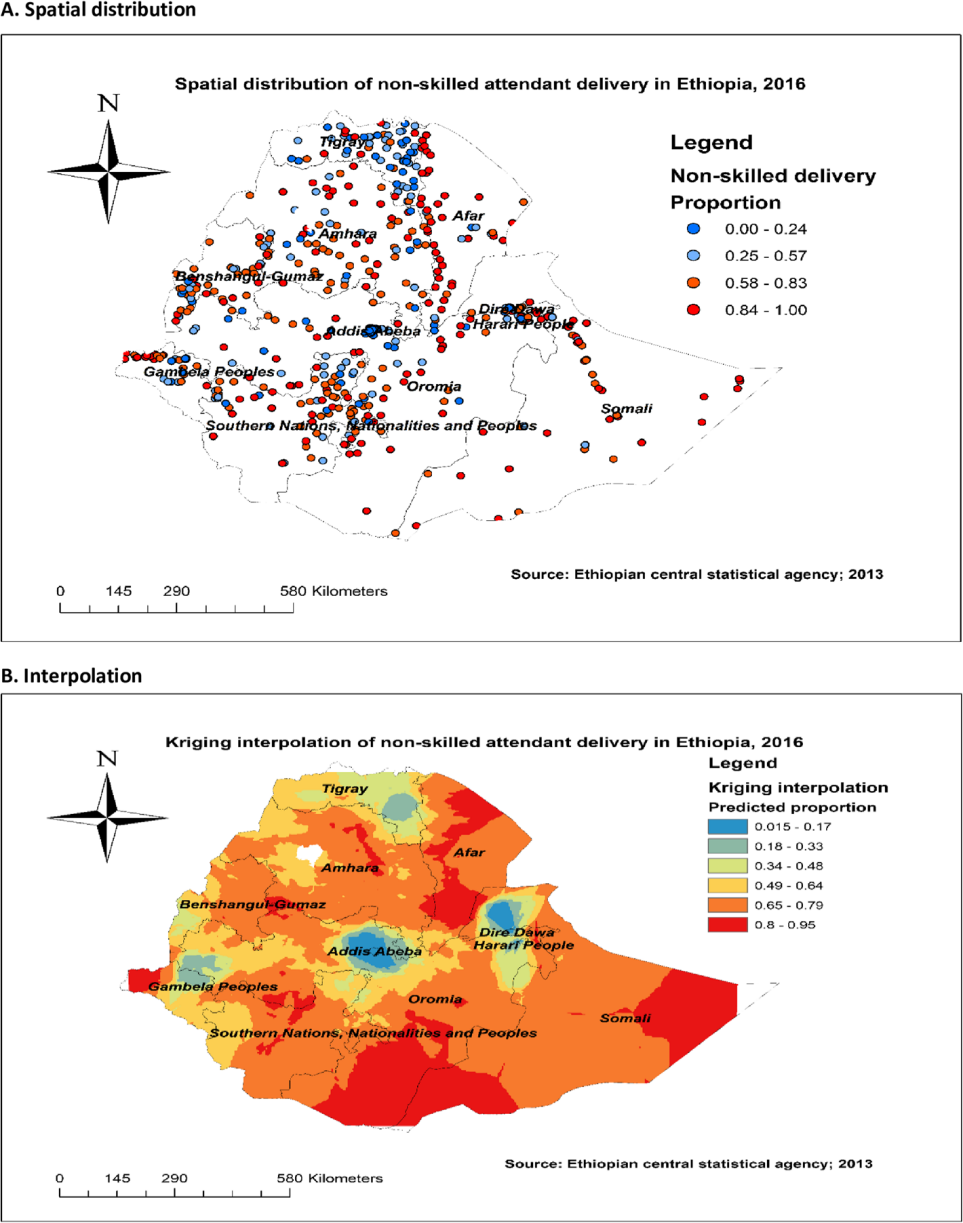
Spatial distribution and interpolation of non-skilled birth attendant delivery in Ethiopia using 2016 EDHS data, Map produced using ArcGIS version 10.3

Figure 1a showed that spatial variation was found in SBA delivery at regional levels. That is the red dots with a higher proportion of non-SBA delivery and the blue dots with a lower proportion of non-SBA delivery. Figure 1b revealed that central, northeastern, and southern parts of Afar, eastern and southwest of Somali, central Amhara, southwest, and southeast Oromia regions have predicted more non-SBA delivery compared to other regions. In contrast, the predicted lower non-SBA delivery was found in Addis Ababa, Dire Dawa, Harari, southeast of Tigray, and around the border between Oromia and Gambela regions (Fig. 1).

#### Hot spot and cold spot analysis of non-SBA delivery

**Fig. 2:**
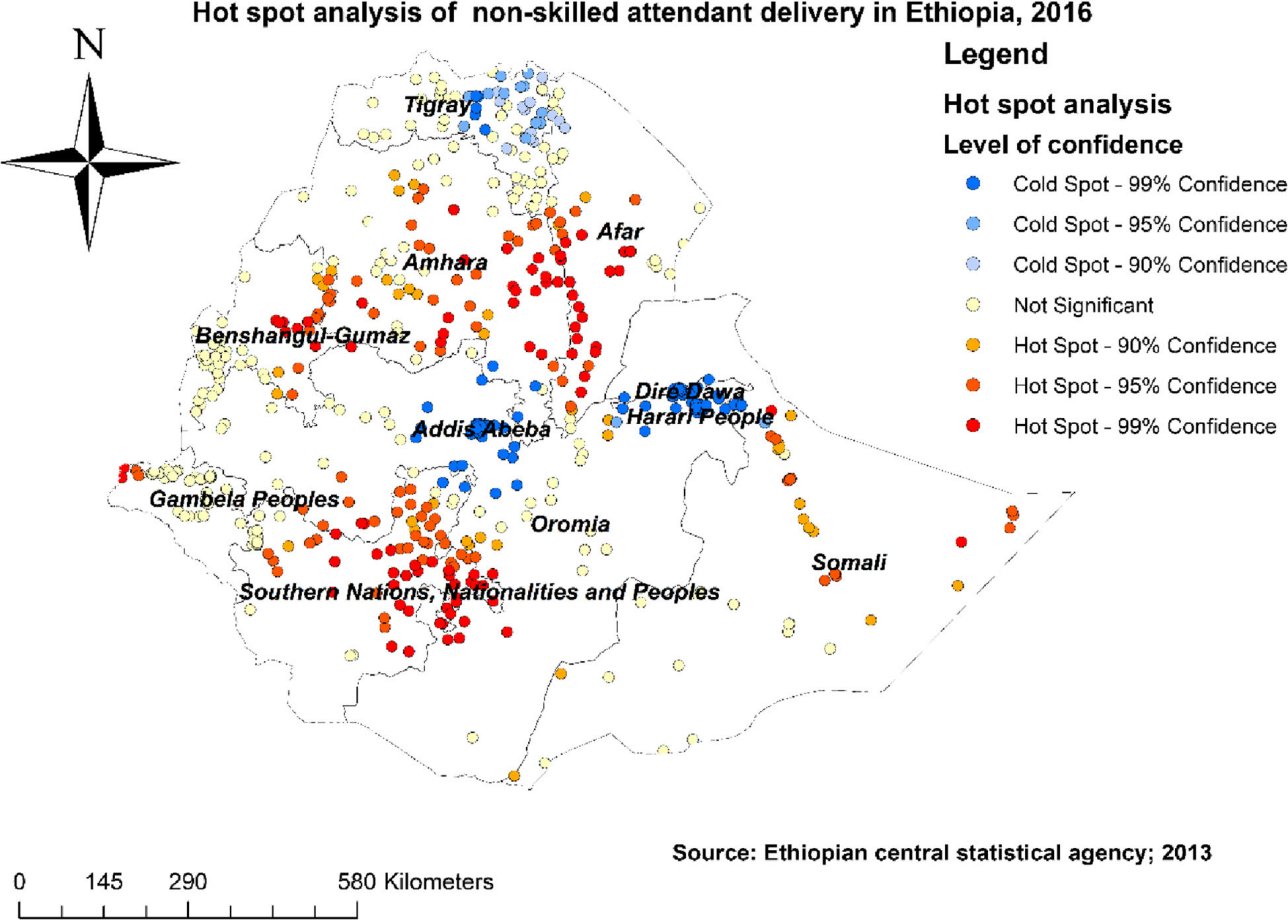
Hotspot and cold spot analysis of non-skilled birth attendant delivery in Ethiopia using EDHS 2016 data, Map produced using ArcGIS version 10.3

The Getis Ord Gi statistical analysis identified the significant hot spot and cold spot areas of non-SBA delivery. The red colors indicated significant hotspot areas (areas with higher rates of non- SBA delivery), which was found in the central and southern eastern parts of Amhara, central and southwestern Afar, northern and eastern part of SNNPR, border of southwestern Oromia, the western part of Gambela, the eastern part of Benishangul, and Somali regions. In contrast, the blue color indicated areas with significant lower rates of non-SBA delivery (cold spot areas) that were found in Addis Ababa, Dire Dawa, Harari, and central and eastern Tigray (Fig. 2).

#### Spatial SaTScan analysis of non-SBA delivery (Bernoulli based model)

The SaTScan analysis detected 288 statistically significant clusters with a high rate of non- SBA delivery. Of these, 104 clusters were the most likely (primary) clusters and 184 were secondary clusters. The primary clusters’ spatial window (the most likely cluster with maximum LLR) was located in southeastern Oromia, the eastern border of SNNPR, and almost the entire Somalia regions, centered at 4.180558 N, 42.052871 E of geographic location with 567.56 km radius, and LLR of 103.49, at *p* < 0.001. This revealed that women within the spatial window had 1.25 times higher risk of non- SBA delivery than women outside the window. The secondary clusters’ scanning window was located in Afar, most areas of Amhara, and the border of Gambela (Additional file 1, Fig. 3).

**Fig. 3:**
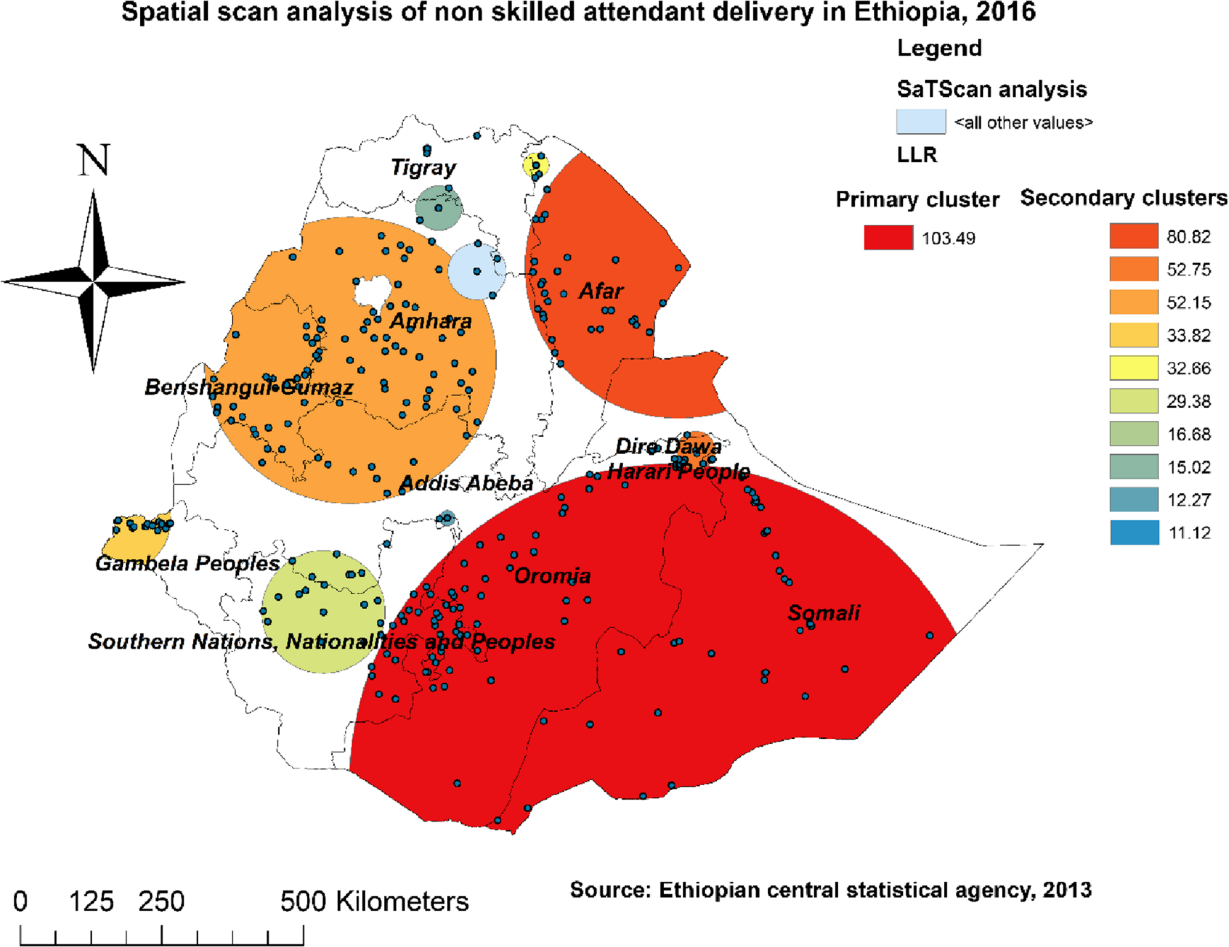
SaTScan analysis of non-skilled birth attendant delivery in Ethiopia, Map produced using ArcGIS version 10.3

### Factors affecting the spatial variation of non-SBA delivery (modeling spatial relationships)

#### Ordinary least square regression

As shown in Table 3 the OLS model explained about 73.9% (Adjusted R square = 0.739) of the variation in non-SBA. In addition, all requirements of the OLS method were fulfilled. The coefficients represent the strength and the type of each explanatory variable and the non-SBA. Since the Koenker (BP) statistic was significant, we used the robust probability to determine the statistical significance of the coefficients and all the coefficients were statistically significant (*p* < 0.01). In addition, the Joint Wald statistic was statistically significant (*p* < 0.01) and these revealed the overall model was significant. Table 3 also revealed no Multicollinearity between explanatory variables (Variance inflation factor (VIF) < 7.5). In addition, the Jarque-Bera statistic was non-significant (*p* = 0.103) indicating the model residuals were normally distributed after rejecting the null hypothesis stating the residuals are normally distributed (Table 3). Moreover, the Spatial Autocorrelation test (Moran’s I = 0.11, *P* < 0.01) revealed that residuals were spatially autocorrelated.

**Table 3:** Summary of OLS results and OLS diagnostics for non-SBA delivery in Ethiopia, EDHS 2016

Variable	Coefficients	Standard error	t-statistics	Probability	Robust standard error	Robust t-statistic	Robust probability	VIF
Intercept	0.05	0.028	1.66	0.097	0.026	1.80	0.073	–
Women with no education	0.31	0.034	9.14	< 0.01	0.041	7.58	< 0.01	2.38
Women from poor household wealth status	0.14	0.032	4.40	< 0.01	0.033	4.20	< 0.01	2.69
Women with no health insurance	0.05	0.019	2.34	0.02	0.020	2.24	0.026	1.03
Women with no media exposure	0.34	0.036	9.51	< 0.01	0.043	7.95	< 0.01	2.92
Women with distance from the health facility as a big problem	0.16	0.028	5.63	< 0.01	0.030	5.39	< 0.01	1.69
Women with parity of one	−0.19	0.050	−3.79	< 0.01	0.045	−4.16	< 0.01	1.48
OLS diagnostics								
Number of observation	622		Akaike’s Information Criterion (AICc)				− 323.96	
Multiple R-squared:	0.742		Adjusted R-Squared				0.739	
Joint F-statistic:	294.10		Prob(> F), (6615) degrees of freedom				< 0.01	
Joint Wald Statistic	3317.02		Prob(> chi-squared), (6) degrees of freedom:				< 0.01	
Koenker (BP) Statistics	21.08		Prob(> chi-squared), (6) degrees of freedom:				< 0.01	
Jarque-Bera Statistics:	4.55		Prob(> chi-squared), (2) degrees of freedom:				0.103	

#### Geographically weighted regression

Since the OLS regression only identified the predictors of non-SBA delivery, it is a global model that assumes the relationship between each explanatory variable and non-SBA delivery is stationary across the study area, we conducted GWR to improve the model in case of non-stationarity between predictors and non-SBA. As shown in Tables 3 and 4, the higher adjusted R square, the lower Akaike’s Information Criterion (AICc) value obtained from the GWR model (as compared to the OLS model) helps us to move from a global model (OLS) to a local regression model (GWR). That is conducting the GWR improves the model (Tables 3 and 4).

**Table 4:** Geographically weighted regression (GWR) model for non-SBA delivery in Ethiopia, EDHS 2016

Explanatory variables	No education, poverty, not covered by health insurance, no media exposure, perceiving distance from the health facility as a big problem, Primiparous
Residual squares	18.25
Effective number	40.27
Sigma	0.177
Akaike’s Information Criterion (AICc)	− 365.98
Multiple R-Squared	0.776
Adjusted R-Squared	0.761

**Fig. 4:**
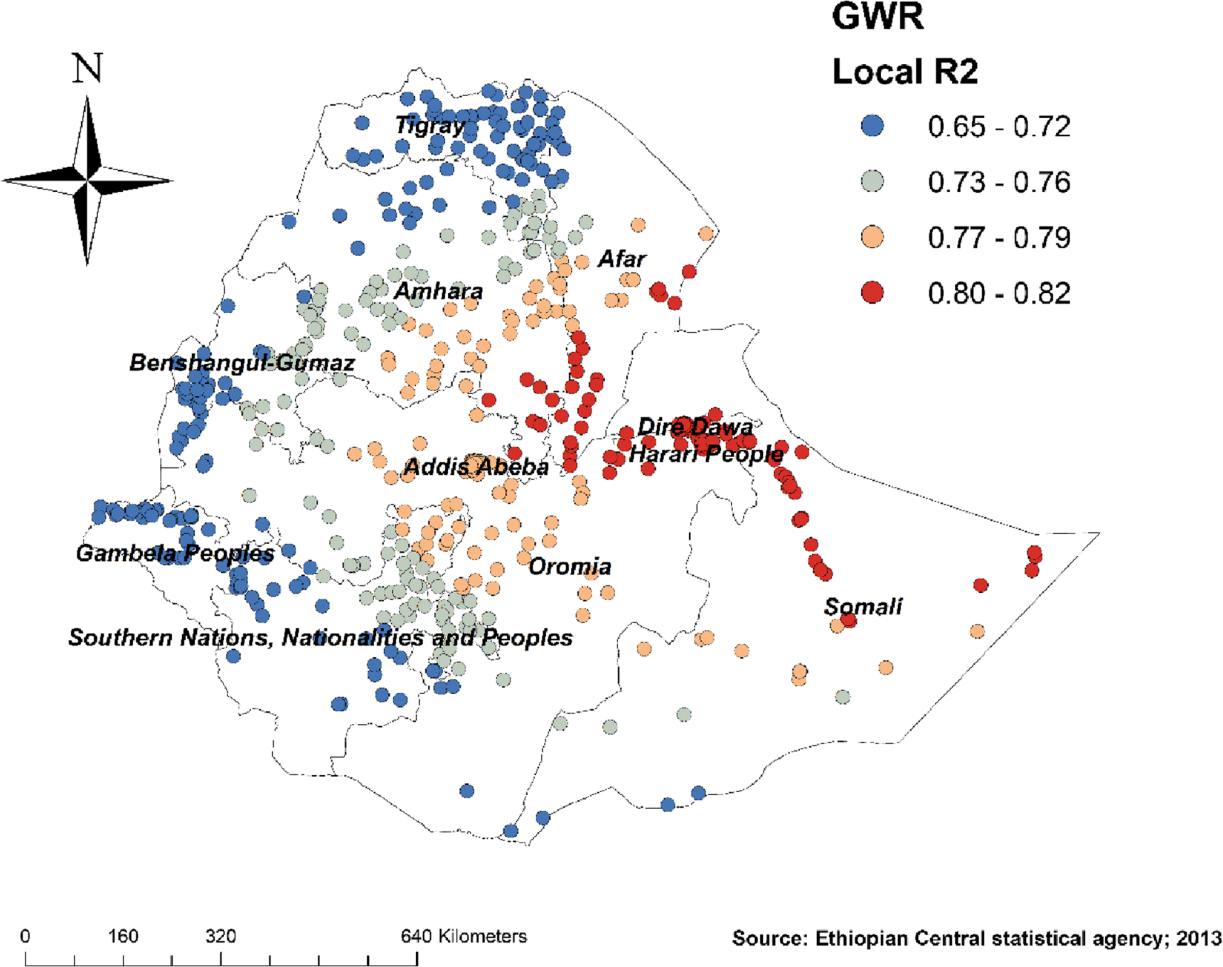
R square for showing model performance for predicting non-SBA delivery in Ethiopia, Map produced using ArcGIS version 10.3

**Fig. 5:**
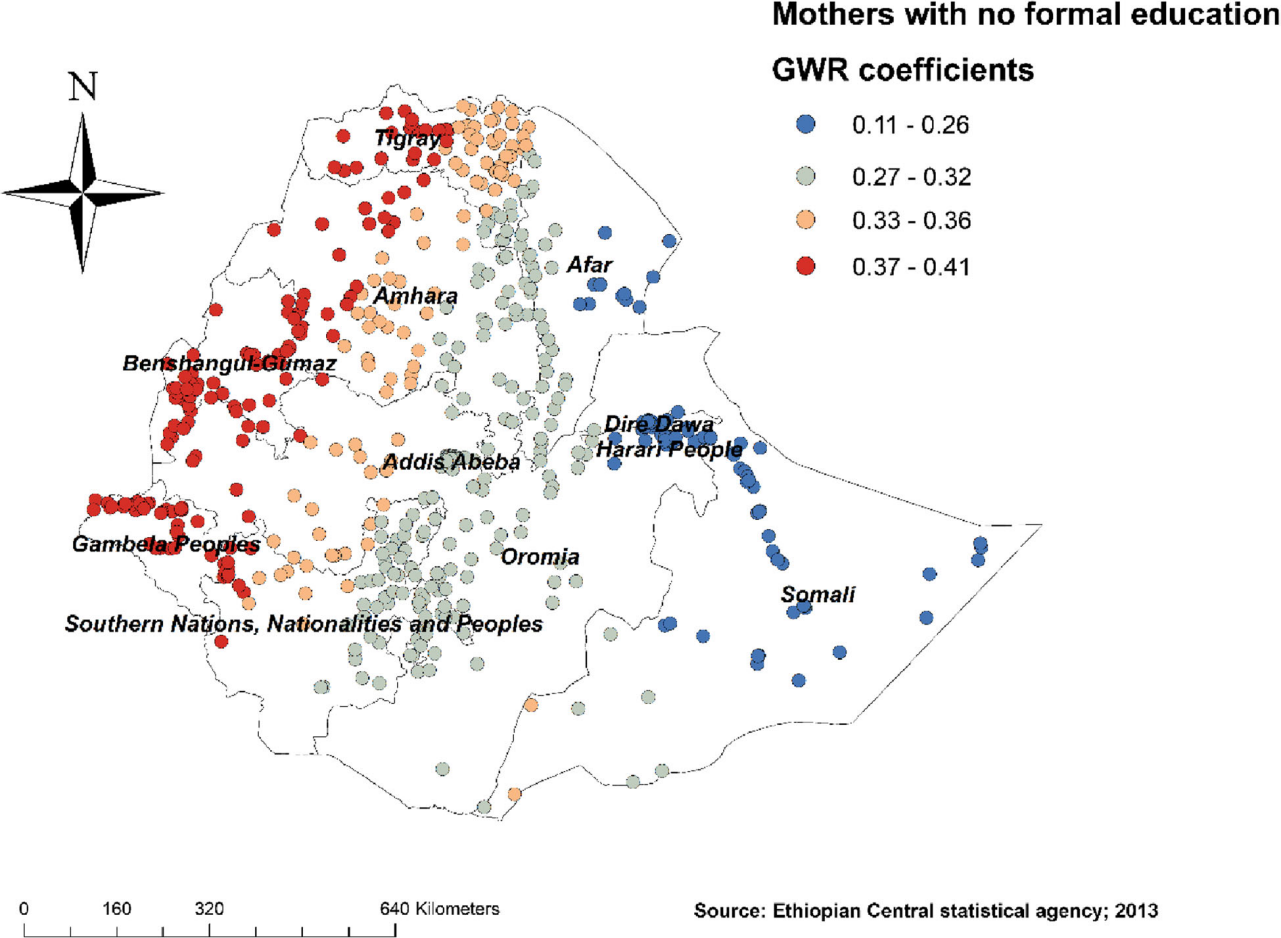
Mothers with no education GWR coefficients for predicting non-SBA delivery in Ethiopia, Map produced using ArcGIS version 10.3

**Fig. 6:**
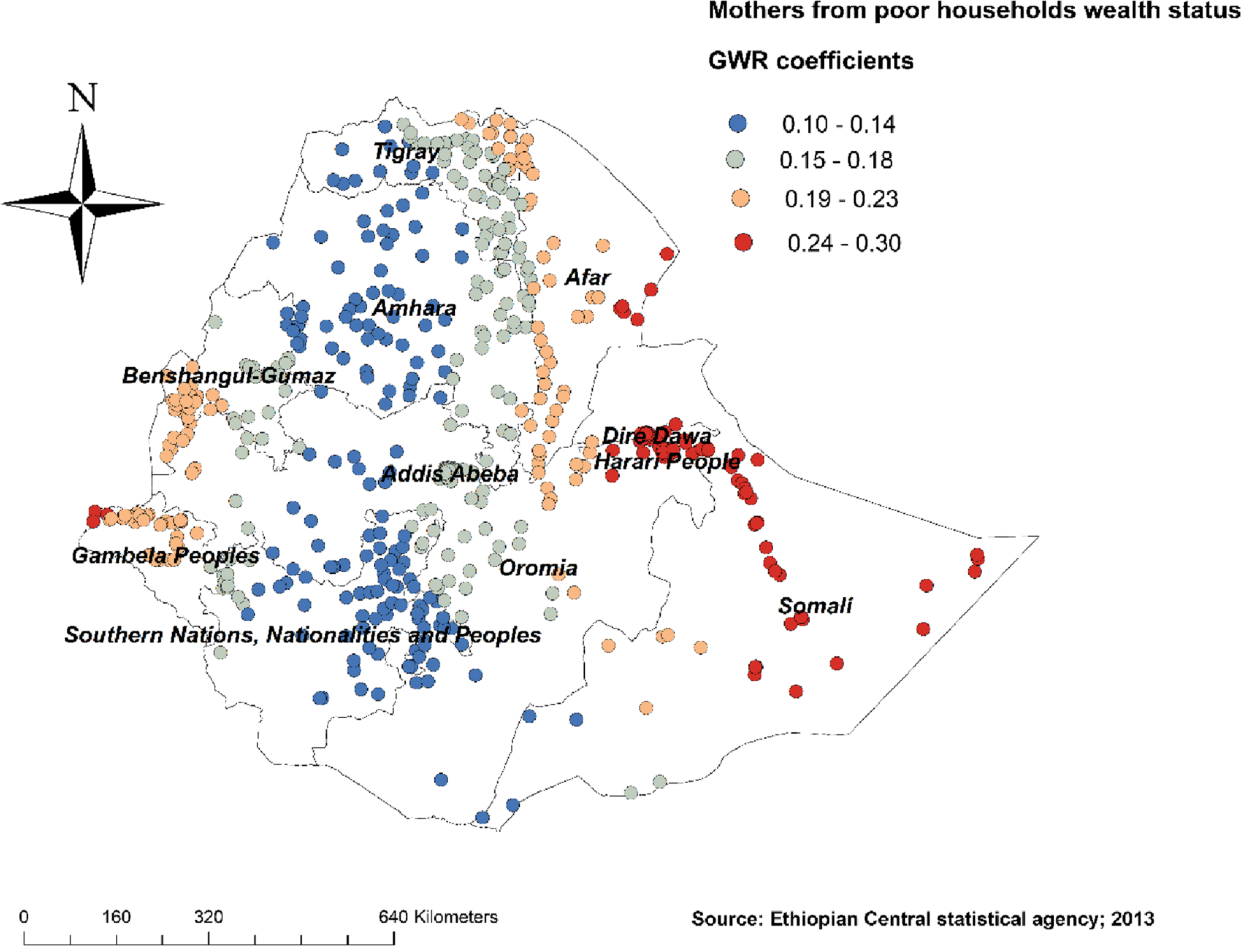
Mothers from poor households GWR coefficients for predicting non-SBA delivery in Ethiopia, Map produced using ArcGIS version 10.3

**Fig. 7:**
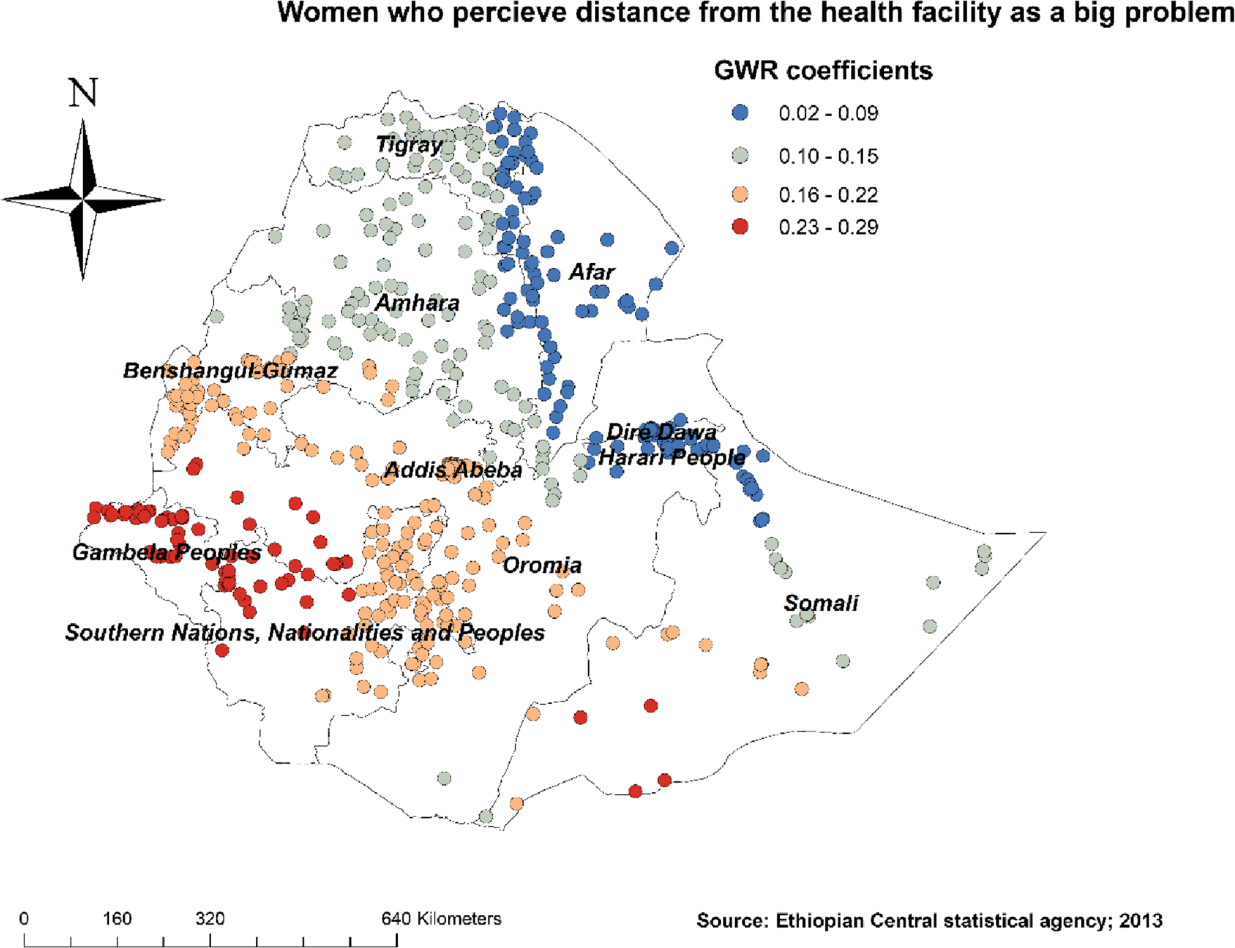
Mothers perceiving distance from the health facility as a big problem GWR coefficients for predicting non-SBA delivery in Ethiopia, Map produced using ArcGIS version 10.3

**Fig. 8:**
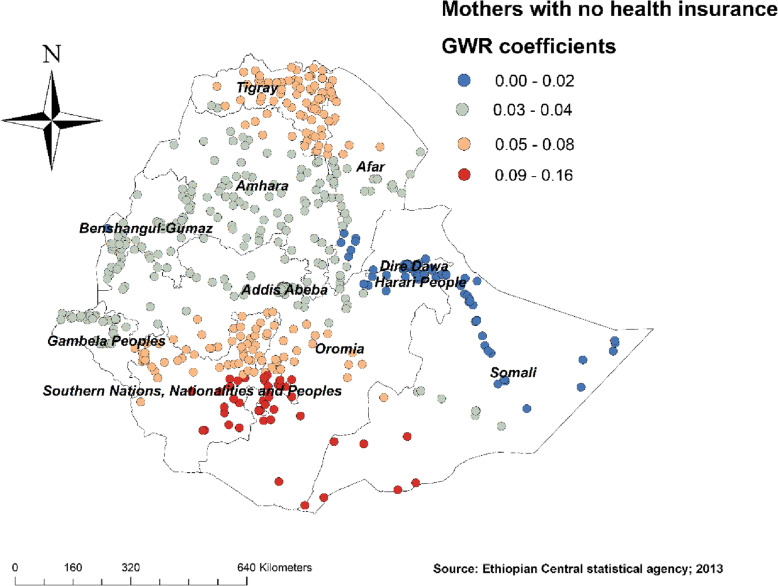
Mothers with no health insurance coverage GWR coefficients for predicting non-SBA delivery in Ethiopia, Map produced using ArcGIS version 10.3

**Fig. 9:**
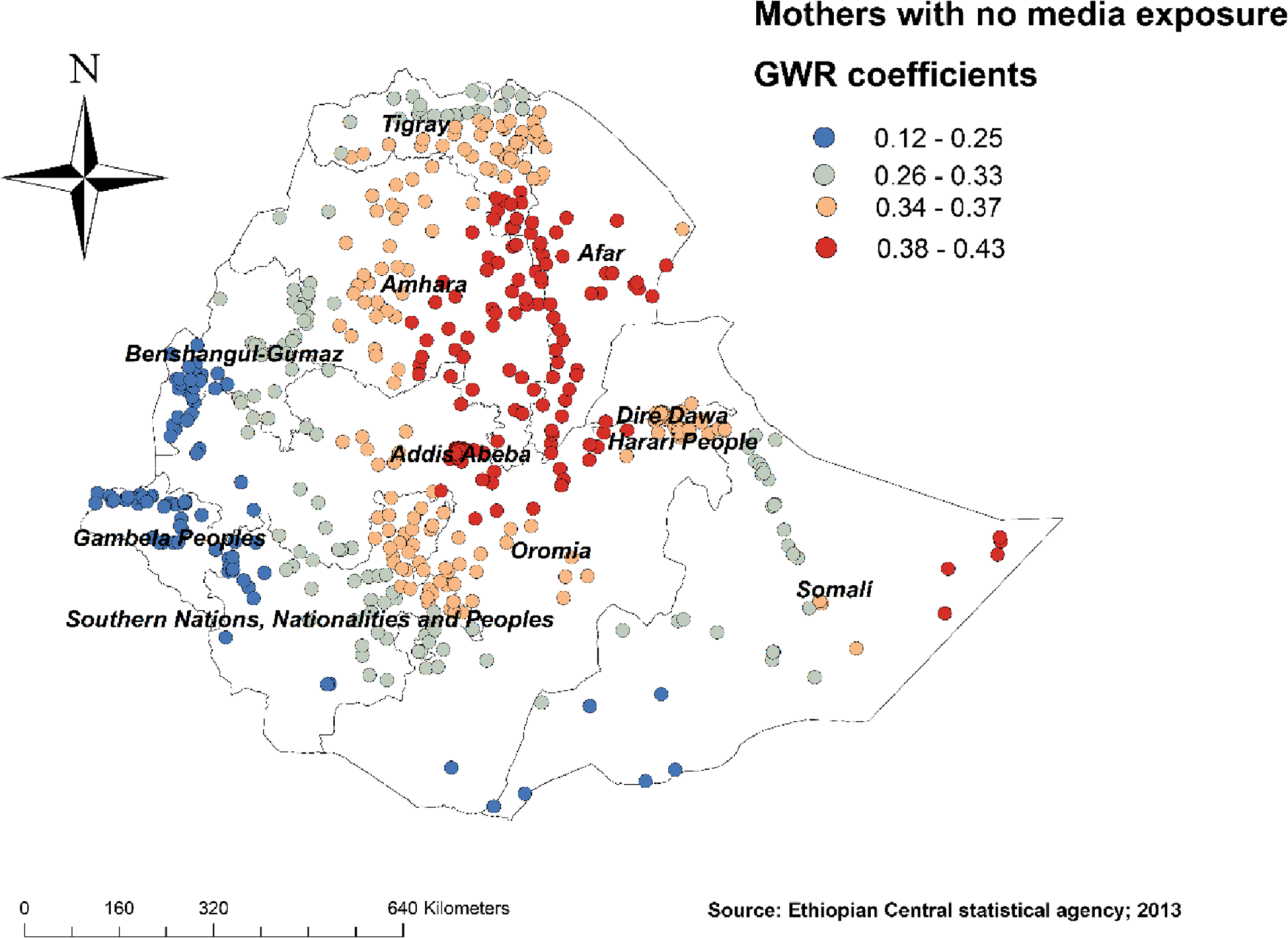
Mothers with no media exposure GWR coefficients for predicting non-SBA delivery in Ethiopia, Map produced using ArcGIS version 10.3

**Fig. 10:**
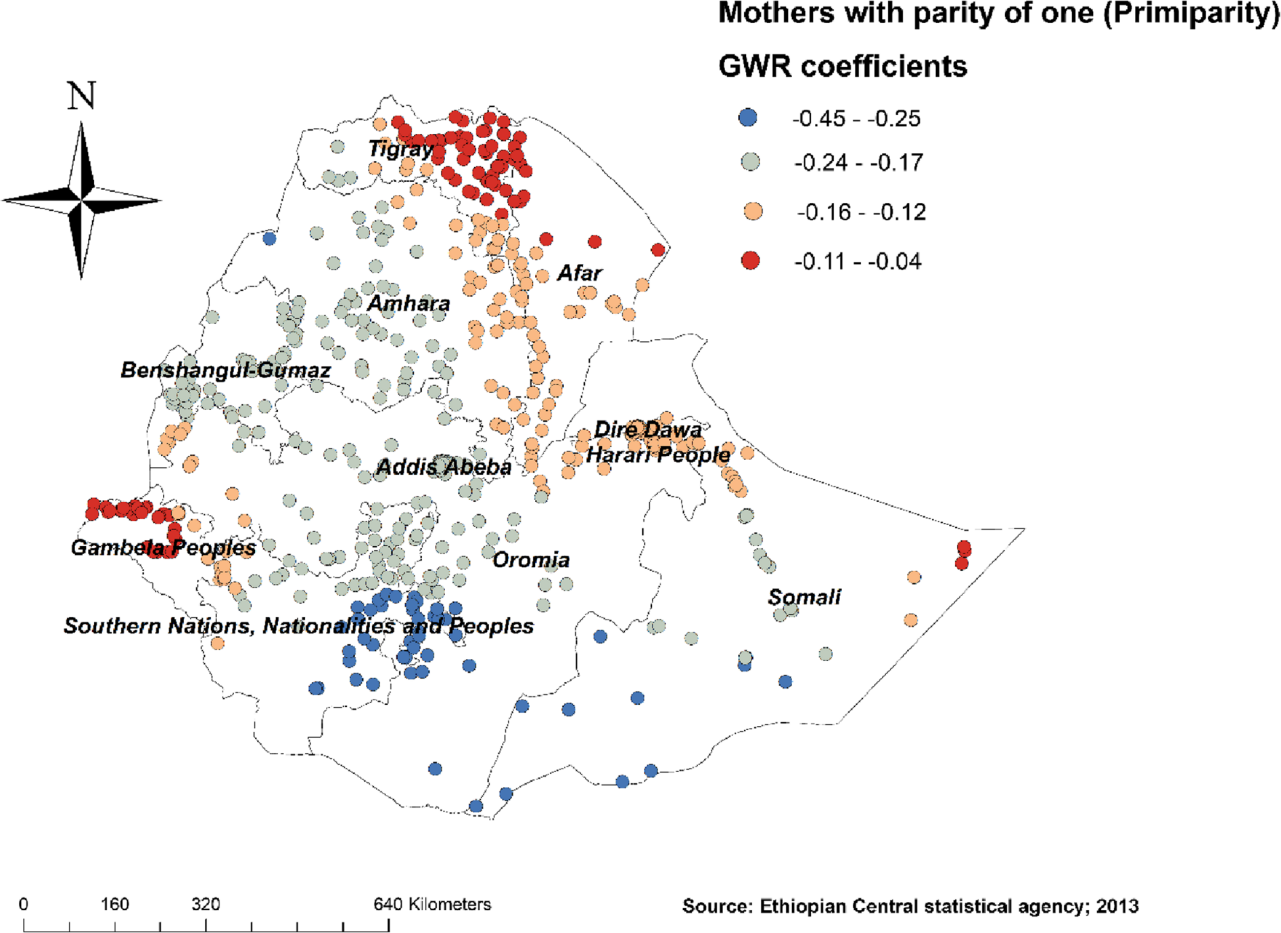
Being Primiparous mothers GWR coefficients for predicting non-SBA delivery in Ethiopia, Map produced using ArcGIS version 10.3

Figure 4 revealed the model performance (local R square) in which it was well performed/explained in southern and eastern parts of Afar, southeastern parts of the Amhara, and northeastern parts of Somalia regions (Fig. 4). Figures 5, 6, 7, 8, 9, and 10 demonstrate the geographical areas where the explanatory variables were strong and weak predictors of non-SBA delivery in Ethiopia. Being mothers with no formal education had a positive relationship with non-SBA delivery. The red-colored clustered points (found in western parts Tigray, Amhara, Benishangul, SNNPR, and entire Gambela) indicate areas where the coefficients were largest, which in turn indicate the strong positive relationship between not attending formal education and non-SBA delivery (Fig. 5). As shown in Fig. 6 being mothers from poor household wealth status showed a strong and positive relationship with non-SBA delivery in western Gambela region, eastern Somalia region, eastern Afar region, Dire Dawa, and Harari (Fig. 6). Being perceiving distance from the health facility as a big problem was also a significant predictor of non-SBA delivery. The strong and positive relationship was found in western Oromia, Gambela, and southwestern Somalia regions (Fig. 7). Regarding health insurance coverage, mothers with no health insurance had a strong and positive relationship with non-SBA delivery in the southwestern Oromia region, southwestern Somalia region, and the eastern part of SNNPR (Fig. 8). As shown in Fig. 9, mothers with no media exposure had a strong and positive relationship with non-SBA delivery in some parts of Afar, Amhara, Addis Ababa, Oromia, and Somalia regions (Fig. 9). Moreover, mothers with parity of one (being Primiparous) had a strong and negative relationship with non-SBA delivery in Tigray, Gambela, and some parts of Afar and Somalia regions (Fig. 10).

### Multilevel analysis

#### Random effect model and model fitness

In the null model, the ICC value was 0.648 indicating that about 64.8% of the total variation on SBA delivery was attributable to differences between communities/clusters. The MOR in the null model (MOR = 10.4) revealed that the likelihood of SBA delivery was different between clusters. Similarly, the highest PCV (85%) in the final model (model III), revealed that 85% of the variability in delivery by SBA was explained by the final model (the combined individual and community level factors). Regarding model fitness, the final model (incorporate both individual and community level factors) was the best-fitted model for this data since it had the lowest deviance (Table 5).

**Table 5:** Random effect and model fitness showing the influence of community characteristics on SBA delivery

Parameter	Null model	Model I	Model II	Model III
Community-level variance (SE)	6.066(0.522)	1.471(0.149)	0.999(0.103)	0.909(0.098)
Log likelihood	− 4987.26	− 4485.29	− 4551.55	− 4289.99
LR test	*X*^*2*^ = 3571.78; *P* < 0.001	*X*^*2*^ = 845.52; *P* < 0.001	*X*^*2*^ = 549.32; *P* < 0.001	*X*^*2*^ = 458.11; *P* < 0.001
Deviance	9974.52	8970.58	9103.10	8579.98
MOR	10.38 (8.59–12.75)	3.24(2.90–3.66)	2.52(2.31–2.79)	2.47(2.25–2.73)
PCV	Ref	0.748	0.844	0.851
ICC	0.648	0.317	0.224	0.215

#### Factors associated with SBA delivery (fixed-effect analysis)

First bi-variable multilevel logistic regression analysis was done and those variables with *p* < 0.2 (all variables had *p* < 0.2 in the bivariable analysis) were considered for multivariable analysis. In the multivariable multilevel analysis: women’s education level, parity, health insurance, wealth index, community poverty level, perception of distance from the health facility, residence, region, and community childcare burden were significantly associated with SBA delivery (*P* ≤ 0.05) (Table 6).

**Table 6:** Multivariable multilevel logistic regression analysis for factors associated with skilled birth attendant delivery, Ethiopia, EDHS 2016

Variables	Null model	Model I (AOR 95%CI)	Model II (AOR 95%CI)	Model III (AOR 95%CI)
Maternal age (years)				
15–24		1.00		1.00
25–34		1.10(0.92–1.31)		0.98(0.82–1.17)
35–49		1.38(1.10–1.75)		1.13(0.89–1.43)
Maternal education				
No formal Education		1.00		1.00
Primary		1.88(1.63–2.18)		1.68(1.45–1.94)
Sec & above		4.96(3.86–6.38)		3.79(2.94–4.89)
Religion				
Orthodox Christian		1.00		1.00
Muslim		0.65(0.52–0.81)		1.06(0.82–1.39)
Protestant		0.59(0.46–0.76)		0.75(0.56–1.00)
Other		0.46(0.28–0.77)		0.61(0.37–1.01)
Parity				
Primiparous		1.00		1.00
Multiparous		0.40(0.32–0.54)		0.41(0.31–0.53)
Grand multiparous		0.28(0.20–0.40)		0.31(0.22–0.44)
Birth order				
1st order		1.00		1.00
2nd order		1.10(0.86–1.42)		1.13(0.88–1.46)
3rd order		0.77(0.59–1.01)		0.81(0.62–1.07)
4th and above order		0.94(0.70–1.27)		1.03(0.76–1.39)
Covered by health insurance				
No		1.00		1.00
Yes		1.68(1.19–2.39)		1.67(1.17–2.37)
Media exposure				
No		1.00		1.00
Yes		1.24(1.07–1.43)		1.14(0.98–1.32)
Wealth index				
Poorest		1.00		1.00
poorer		1.82(1.51–2.20)		1.57(1.30–1.90)
Middle		2.00(1.64–2.45)		1.63(1.32–2.00)
Richer		2.40(1.93–2.99)		1.79(1.43–2.25)
Richer		9.13(7.11–11.73)		2.56(1.92–3.41)
Perception of distance from the health facility				
Big problem			1.00	1.00
Not a big problem			1.49(1.31–1.70)	1.36(1.19–1.55)
Residence				
Urban			1.00	1.00
Rural			0.24(0.16–0.34)	0.37(0.25–0.55)
Region				
Afar			1.00	1.00
Tigray			9.60(5.86–15.74)	8.17(4.72–14.14)
Amhara			1.79(1.09–2.95)	1.48(0.87–2.55)
Oromia			1.41(0.86–2.32)	1.25(0.76–2.08)
Somali			1.86(1.15–3.04)	1.98(1.22–3.22)
Benishangul			2.97(1.78–4.97)	2.80(1.65–4.74)
SNNPR			2.54(1.55–4.17)	2.62(1.53–4.50)
Gambela			2.53(1.49–4.31)	2.56(1.44–4.56)
Harari			4.56(2.57–8.11)	3.70(2.07–6.60)
Addis Ababa			14.90(6.89–30.90)	8.67(4.01–18.73)
Dire Dawa			6.45(3.60–11.56)	5.33(2.96–9.61)
Community-level of women education				
Lower			1.00	1.00
High			1.73(1.34–2.23)	1.27(0.98–1.65)
Community poverty level				
Low			1.00	1.00
Medium			0.53(0.39–0.73)	0.65(0.47–0.90)
High			0.35(0.25–0.50)	0.51(0.36–0.74)
Community level of media exposure				
Low			1.00	1.00
Medium			1.14(0.83–1.57)	1.09(0.80–1.50)
High			1.49(1.08–2.05)	1.33(0.96–1.84)
Community childcare burden				
Low			1.00	1.00
Medium			0.64(0.47–0.87)	0.76(0.57–1.03)
High			0.44(0.33–0.59)	0.54(0.40–0.72)

*AOR* Adjusted odds ratio, *CI* Confidence Interval

Women who had primary education, and secondary and above education had 1.68 (Adjusted Odds Ratio (AOR) = 1.68; 95% CI: 1.45–1.94) and 3.79 (AOR = 3.79; 95% CI: 2.94–4.89) times higher odds of giving birth by SBA as compared to women who had no formal education. Women who were multiparous and grand multiparous had 59% (AOR = 0.41; 95% CI: 0.31–0.54) and 69% (AOR = 0.31; 95% CI: 0.22–0.44) lower odds of SBA delivery respectively as compared to Primiparous women. Women who were covered by health insurance had 1.67 times (AOR = 1.67; 95% CI: 1.17–2.37) higher odds of SBA delivery. Women from households with poorer, middle, richer and richest wealth status had 1.57 (AOR = 1.57; 95% CI: 1.30–1.90), 1.63 (AOR = 1.63; 95% CI: 1.32–2.00), 1.79 (AOR = 1.79; 95% CI: 1.43–2.25), and 2.56 (AOR = 2.56; 95% CI: 1.92–3.41) times higher odds of SBA delivery respectively. Women who did not perceive distance from the health facility as a big problem had 1.36 times (AOR = 1.36; 95% CI: 1.19–1.55) higher odds of SBA delivery. Women from rural areas had 63% (AOR = 0.37; 95% CI: 0.25–0.54) lower odds of SBA delivery as compared to their counterparts. Regarding community poverty level, women residing in communities with medium and higher poverty level had 35% (AOR = 0.65; 95% CI: 0.47–0.90) and 49% (AOR = 0.51; 95% CI 0.36–0.74) lower odds of giving birth by SBA as compared to women residing in communities with low poverty level respectively. Similarly, women residing in communities with higher childcare burden had 46% (AOR = 0.54; 95% CI: 0.40–0.72) lower odds of SBA delivery compared to women residing in communities with lower childcare burden (Table 6).

## Discussion

This study aimed to determine the spatial variations of SBA delivery and its associated factors in Ethiopia using advanced statistical models. The finding of this study showed that SBA delivery had significant spatial variation in Ethiopia. The spatial scan statistics detected a total of 104 statistically significant primary clusters with a high prevalence of non- SBA delivery. Significant hotspot areas of non-SBA delivery (primary clusters) were observed in the eastern border of SNNPR, Somali, and southeastern Oromia region, while the safest regions who had a higher proportion of SBA delivery were Addis Ababa, Dire Dawa, and Tigray. Other studies conducted in developing countries also pointed out the significant regional variations in the use of SBA delivery [26, 27]. This might be because of inaccessible health facilities as well as inequalities in the distribution of scarce resources like skilled health professionals to remote/border areas of Ethiopia. In addition, this might be due to the socio-cultural and socioeconomic differences between women in different regions. Moreover, mothers from border areas might have limited access to information regarding maternal health services and other services like access to school/education.

The GWR analysis revealed a positive relationship (both strong and weak positive relationships) between mothers having no formal education, mothers from poor household wealth status, those perceiving distance from the health facility as a big problem, being not exposed to media, and being not covered by health insurance with non-SBA delivery in different regions of Ethiopia. However, being Primiparous mother had a negative relationship, both strong and weak, with non-SBA delivery in different regions of Ethiopia. The findings (the predictor variables found in the GWR analysis) were similar to the multilevel analysis conducted in this study. The authors recommend further investigations by incorporating other predictors such as behavioral factors and health service-related factors like quality of maternal health service delivery.

After controlling for clustering/random effect, this study also identified both individual and community level factors that have a significant association with SBA delivery. Among individual factors; women’s education, parity, health insurance coverage, and wealth index were significantly associated with SBA delivery. Residence, region, perception of distance from the health facility, community poverty level, and community child care burden were among the community level factors which were significantly associated with SBA delivery.

The finding of this study revealed that women with primary and higher education had a positive significant association with SBA delivery. This result is consistent with the findings of several studies elsewhere [23, 25, 26, 43, 44]. The possible explanation could be: educated women have increased awareness about their health to seek out and utilize modern health care services such as SBA delivery.

In this study, a negative association between the uptake of SBA delivery and parity was observed. Multiparous and grand multiparous women had lower odds of SBA delivery and this finding is congruent with a study done in Nigeria [27]. This might be because women with higher parity might not have any complications before and take childbirth as a natural process or might have bad contact with a health professional previously, so they may prefer delivering without SBA [45, 46].

Those women who were covered by health insurance had higher odds of SBA delivery. This finding is in line with studies conducted in Ghana [30], Indonesia [28], and Kenya [47]. This might be because if a woman is covered by health insurance, they sense free and seek any medical care frequently. Also, if a woman is covered by health insurance there might not be difference between those mothers from rich households and poor households in using SBA, and this is supported by our study in which wealth status is a significant factor associated with SBA.

Moreover, a consistent positive association between SBA delivery and the household’s wealth status was observed. Mothers from poorer to richest households had higher odds of SBA delivery compared to poorest households and this is in line with different studies conducted in different countries [23, 25, 28, 30]. Even though the utilization of maternal health services in Ethiopia does not need payment, this could not give a guaranty for the use of SBA delivery. This is because of the presence of other costs such as time and transportation that can determine SBA delivery utilization.

The study further found that community-level characteristics are crucial for the use of SBA delivery in Ethiopia. Women residing in communities with medium and higher poverty levels had lower odds to give birth by SBA as compared to women residing in communities with low poverty level. This finding is supported by a study done in Nigeria [27]. This is due to communities with higher poverty level might not be able to pay for health insurance [48] and the actual enrollment in health insurance is directly associated with SBA in our study. Similarly, women residing in communities with higher childcare burden had a negative association with SBA and this finding is in line with studies in Ethiopia [28] and Nigeria [27]. This might be due to a high childcare burden that may have problems related to cost and time for carrying children or family in general, which may prevent mothers from seeking and utilizing maternal health services like SBA delivery [49].

Women from rural areas were less likely to give birth by a skilled birth attendant. This finding is supported by different studies done in Bangladesh [25] and Ethiopia [28, 44]. This could be because rural women may not have access to education regarding maternal health services. Moreover, they may face challenges in accessing health facilities that might lead to very low antenatal care utilization, and then lower odds of SBA delivery.

Furthermore, participants who perceived distance from the health facility as a big problem had lower odds of skilled attendant delivery. This is in line with studies done in Ethiopia [35] and rural Nigeria [27]. This is due to the fact that distance from health facilities and lack of transport are important factors to prevent mothers from seeking and utilizing skilled maternity care services [50–52].

The main strength of this study was the use of nationally representative data with a large sample. The other strength was that we used multilevel analysis that account the correlated nature of the data. The use of spatial analysis including modeling spatial relationships using GWR was also another strength of this study. However, this study is not free from limitations. Medical and health facility-related factors that might influence the SBA delivery among reproductive-age women were not assessed (not collected in the survey). Besides, since the outcome was based on the maternal report, there might be a lapse of memory or recall bias. Also, due to the cross-sectional nature of the data, it is difficult to establish a temporal relationship between the dependent variable and the explanatory variables.

## Conclusion

In Ethiopia, SBA delivery had spatial variations across the regions. Local clusters of areas with a low proportion of SBA delivery were detected in the eastern border of SNNPR, Somali, and southeastern Oromia regions. In the GWR analysis being mothers having no formal education, mothers from poor household wealth status, those perceiving distance from the health facility as a big problem, being not exposed to media, and being not covered by health insurance increased the risk of non-SBA delivery. However, being Primiparous decreases the risks of non-SBA delivery in different regions of Ethiopia. In the multilevel analysis both individual and community level factors were associated with SBA delivery. Among individual-level factors, mothers with primary and above education, being multi and grand multiparous, those women covered with health insurance, and wealth status were significantly associated with SBA delivery. Besides, perception of distance from the health facility as a big problem, rural residence, medium and higher community poverty level, and higher community childcare burden are among community-level factors that were significantly associated with SBA delivery. Therefore, areas with non-skilled birth attendant delivery and mothers who had no formal education, not health insured, mothers from poor households and communities, Primiparous women, mothers from remote/rural areas, and mothers from communities with higher childcare burden could get special attention in terms of allocation of resources including skilled human power, and improved access to health facilities.

## Supplementary information

**Additional file 1.** Significant clusters of areas with a high proportion of non-SBA delivery among women in Ethiopia.

## Data Availability

All data relevant to the study are available within the manuscript and anyone who wants the data set can get through requesting online using the link (http://www.measuredhs.com).

## Abbreviations

AICc: Akaike’s Information Criterion
AOR: Adjusted Odds Ratio
EAs: Enumeration Areas
EDHS: Ethiopian Demographic and Health Survey
ICC: Intraclass Correlation
LLR: Log likelihood Ratio
MOR: Median Odds Ratio
OLS: Ordinary Least Square
PCV: Proportional Change in Variance
SBA: Skilled Birth Attendant
SNNPR: Southern Nations, Nationalities and Peoples’ Region
WHO: World Health Organization
GWR: Geographically Weighted Regression
