# Supplementary material for: Exploring spatial variations and factors associated with skilled birth attendant delivery in Ethiopia: geographically weighted regression and multilevel analysis
Source: BMC Public Health. 2020 Sep 23;20:1444. doi: 10.1186/s12889-020-09550-3 (PMC7519489; doi:10.1186/s12889-020-09550-3)
Supplement: Supplementary file 1 — Additional file 1. Significant clusters of areas with a high proportion of non-SBA delivery among women in Ethiopia. [file 12889_2020_9550_MOESM1_ESM.docx]

| Numbers of significant clusters detected | Coordinates/radius | population | case | RR | LLR | P-value |
| --- | --- | --- | --- | --- | --- | --- |
| 104 | (4.180558 N, 42.052871 E) / 567.56 km | 2359 | 1839 | 1.25 | 103.4883 | <0.001 |
| 31 | (12.401068 N, 42.163134 E) / 265.14 km | 617 | 541 | 1.36 | 80.81624 | <0.001 |
| 10 | (9.505470 N, 42.438628 E) / 33.79 km | 257 | 238 | 1.42 | 52.7516 | <0.001 |
| 91 | (10.934452 N, 36.945496 E) / 252.94 km | 1434 | 111 | 1.21 | 52.14774 | <0.001 |
| 16 | (8.389747 N, 33.258557 E) / 91.71 km | 285 | 248 | 1.33 | 33.81755 | <0.001 |
| 4 | (14.034142 N, 39.898488 E) / 22.09 km | 106 | 103 | 1.48 | 32.66454 | <0.001 |
| 24 | (6.934084 N, 36.520510 E) / 108.17 km | 401 | 332 | 1.27 | 29.38127 | <0.001 |
| 1 | (9.370004 N, 42.102751 E) / 0 km | 40 | 40 | 1.52 | 16.67808 | <0.001 |
| 3 | (13.351814 N, 38.353591 E) / 39.54 km | 77 | 71 | 1.4 | 15.01696 | <0.001 |
| 2 | (8.425886 N, 38.488143 E) / 13.52 km | 46 | 44 | 1.45 | 12.27122 | 0.004 |
| 2 | (14.293660 N, 38.170336 E) / 7.95 km | 49 | 46 | 1.43 | 11.12224 | 0.014 |
